# Turmeric and Curcumin—Health-Promoting Properties in Humans versus Dogs

**DOI:** 10.3390/ijms241914561

**Published:** 2023-09-26

**Authors:** Jagoda Kępińska-Pacelik, Wioletta Biel

**Affiliations:** Department of Monogastric Animal Sciences, Division of Animal Nutrition and Food, West Pomeranian University of Technology in Szczecin, Klemensa Janickiego 29, 71-270 Szczecin, Poland

**Keywords:** antioxidant potential, *Curcuma longa* L., diferuloylmethane, diseases, pharmacological activities, rhizome, SARS-CoV-2

## Abstract

The growing popularity of the use of nutraceuticals in the prevention and alleviation of symptoms of many diseases in humans and dogs means that they are increasingly the subject of research. A representative of the nutraceutical that deserves special attention is turmeric. Turmeric belongs to the family *Zingiberaceae* and is grown extensively in Asia. It is a plant used as a spice and food coloring, and it is also used in traditional medicine. The biologically active factors that give turmeric its unusual properties and color are curcuminoids. It is a group of substances that includes curcumin, de-methoxycurcumin, and bis-demethoxycurcumin. Curcumin is used as a yellow-orange food coloring. The most important pro-health effects observed after taking curcuminoids include anti-inflammatory, anticancer, and antioxidant effects. The aim of this study was to characterize turmeric and its main substance, curcumin, in terms of their properties, advantages, and disadvantages, based on literature data.

## 1. Introduction

**Figure 1:**
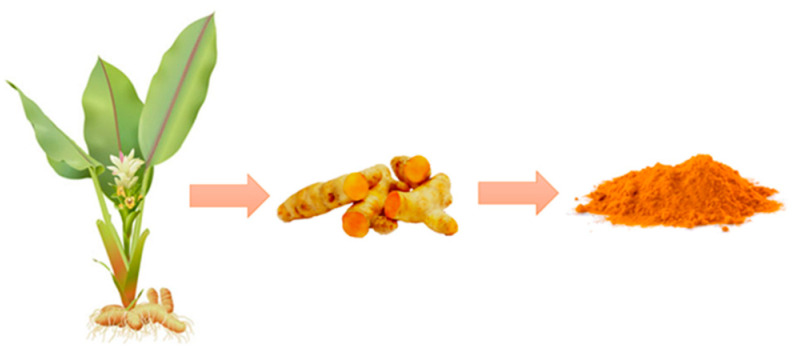
Turmeric—plant, rhizome, and spice.

Up to 41,000 papers until 2023 have been published about curcumin and turmeric [1]. Turmeric is one of the species of the genus *Curcuma* (*Curcuma* L.) of the family Zingiberaceae [2,3]. Turmeric (*Curcuma longa* L.) is also called Zohara turmeric, turmeric long, Indian turmeric, and Indian saffron. It is cultivated in many countries with a tropical climate, which is favorable to the growth of this plant. Turmeric grows wild in India, Southeast Asia, and Indonesia [4,5]. It is characterized by its versatile application, as it is used as a spice, dye, cosmetic, and for decorative purposes, in addition to being a medicinal herbaceous plant. Like chili, turmeric is commonly used in Asian cuisine to impart a yellow color and flavor to foods, and it is also used as a preservative [6] (Figure 1). The aim of this study was to characterize turmeric and its main substance, curcumin, in terms of their properties, advantages, and disadvantages, based on literature data.

Various parts of the plant (rhizomes, leaf, stem, flower, and herbaceous material) are used, mainly in traditional medicine, but the most important seems to be the rhizome [7]. It is characterized by its high nutritional value [8,9,10,11] (Table 1). Research has shown that the chemical composition of the rhizome depends on various factors, including the climatic conditions in which turmeric grows. The nutritional value also depends on the subsequent processing of the raw material [12]. Research has also shown that the in vitro mineral nutrition of this plant influences the production of volatile compounds in the rhizomes after transfer to the greenhouse [13]. In a previous study, fertilization treatments during the growth period in the greenhouse increased the content of volatile substances in rhizomes. Phytochemical concentrations were significantly multiplied as a result of primary metabolism in the ex vitro growth of rhizomes during greenhouse cultivation.

Turmeric is characterized by a high content of total carbohydrates and protein. It is rich in essential amino acids [8,14,15] (Table 2), but not as much as other *Zingiberaceae* species [16]. The rhizome contains macro- and microelements, such as calcium, potassium, sodium, and iron [14,17,18] (Table 3 and Table 4). Certain vitamins are also present (Table 5) [14,19,20]. The turmeric rhizome is a valuable source of active substances—polyphenolic compounds known as curcuminoids (Table 6) [21,22,23]. Curcuminoids include curcumin, desmethoxycurcumin, bisdemethoxycurcumin, and cyclocurcumin. Curcumin (diferuloylmethane) is the major quantitative and biologically important curcuminoid found in turmeric [24,25,26]. It has been shown that increased temperature reduces the content of these compounds [27]. Other active substances that can be found in the turmeric plant are alkaloids, saponins, tannins, phytic acid, and flavonoids [9,28,29] (Table 7).

**Figure 2:**
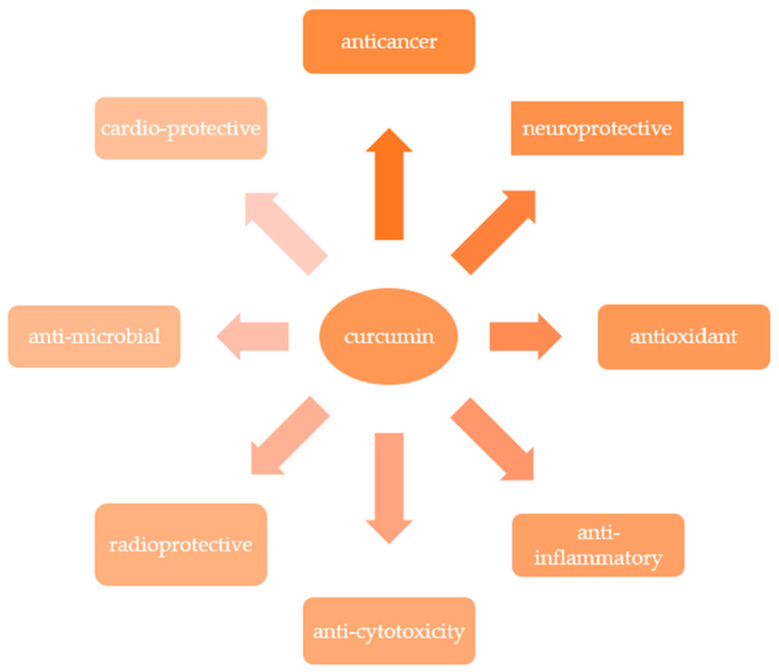
Selected health properties of curcumin. Adapted from [32,33,34,35,36].

Curcuminoids are appreciated all over the world as a functional food because of their health-promoting properties [24,30,31]. There are several reports in the literature indicating that turmeric is one of the most popular medicinal herbs, with a wide range of pharmacological properties such as anti-inflammatory anticancer, antioxidant, antiproliferative, and antimicrobial properties [32,33,34,35,36] (Figure 2).

Turmeric is a source of essential oils that are responsible for its scent. Terpenoids are the major component in the essential oil of turmeric [25]. The essential oils that contribute to the antioxidant properties in turmeric rhizome are ar-tumerone, curlone, and ar-curcumene [37,38,39] (Table 8).

Turmeric has the Generally Recognized as Safe (GRAS) status. The US Food and Drug Administration (FDA) gives certain substances this designation, which means that the use of this substance as a food additive is recognized by experts as safe for health [40].

## 2. The Main Compound—Curcumin

**Figure 3:**
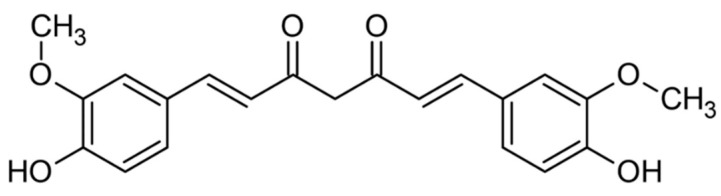
Chemical structure of curcumin.

**Figure 4:**
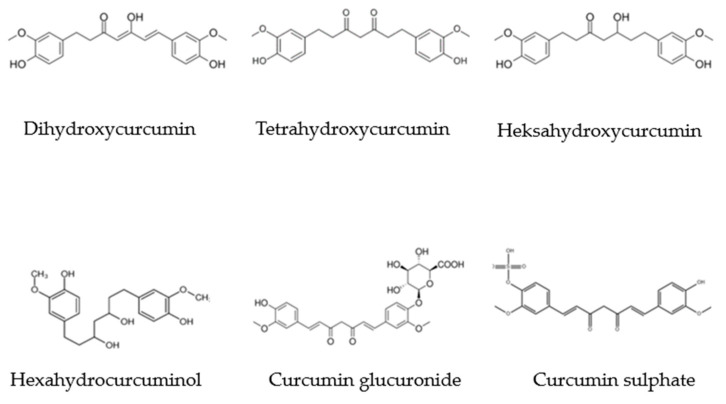
Chemical structure of the main metabolites of curcumin.

The curcumin (Figure 3) molecule (C_21_H_20_O_6_, (1E,6E)-1,7-bis-(4-hydroxy-3-methoxyphenyl) hepta-1,6-dien-3,5-dione) was first isolated from turmeric in 1815 [41]. It is resistant to heating (<120 °C) and the action of acids (stable in the pH range 2.5–6.5). In an acid environment, the color of curcumin is yellow, while in an alkaline it changes to reddish brown. Curcumin is characterized by having good solubility in ethanol, acetone, methanol, and oils, and very low solubility in water [42]. Curcumin metabolites are also important (Figure 4).

According to the markings of the European Union, curcumin is defined as a food additive under the symbol E100, according to the Color Index International—Natural Yellow. In commercial form, it is present as oil with 3–5% curcumin content, oleoresin with 37–55% curcumin content, or purified curcumin with carrier [43]. For food additives, the acceptable daily intake (ADI) of curcumin is determined on the basis of the tests performed. ADI is the amount of a given substance, expressed in mg/kg of human body weight per day, that a person can take from all sources throughout life without harming the organism. In 2004, the FAO/WHO Expert Committee on Food Additives (JECFA) established an ADI of up to 3 mg/kg body weight for curcumin. However, this indicator is exceeded in many European countries [44,45,46]. This should not be of concern to consumers as curcumin has antioxidant, anti-inflammatory, and anticancer effects. Research has also been conducted on its use in the treatment of neurodegenerative diseases. Moreover, it was shown that curcumin can inhibit the spread of parasites, bacteria, and pathogenic fungi [47].

Already in antiquity, curcumin was used as an anti-inflammatory agent and in digestive ailments, such as indigestion, flatulence, and diarrhea [48]. In recent years, much attention has been paid to the use of curcumin in medicine to treat and alleviate the symptoms of diseases related to oxidative stress, inflammation, and many types of cancer [49,50,51,52,53,54,55].

Many years of research have shown that curcumin inhibits the development of diseases, which is directly related to its high antioxidant activity [56]. This compound has a neuroprotective effect in brain injuries and diseases of the nervous system (neurodegenerative diseases), and it has hepatoprotective, anticoagulant, hypoglycemic, and antilipemic effects by reducing total cholesterol and LDL (low-density lipoprotein) fractions and increasing HDL (high-density lipoprotein) fractions and triglycerides [57,58,59].

## 3. Extraction

Curcumin is mainly obtained using two methods: synthesis and extraction from plants. Although synthetic curcumin can be obtained, as has been reported by many authors [60,61], using extraction to obtain curcumin, which is naturally present in plants, is still the most economical method, and various techniques have been reported to efficiently extract curcumin from plant materials. These include conventional methods (e.g., Soxhlet extraction, maceration, and solvent extraction) [62,63] and advanced extraction technologies (e.g., ultrasonically assisted extraction, microwave-assisted extraction, enzyme-assisted extraction, supercritical fluid extraction, etc.) [63,64,65,66,67]. It was stated that the method and type of extraction play the most important role in determining the quantity and quality of bioactive compounds. In order to obtain curcumin of high efficiency and quality, it is necessary to choose appropriate and effective methods and to carry out the process under optimal conditions [68].

The solubility, extraction, and stabilization of the curcumin extracted from *Curcuma longa* L. remain a challenge. The most common problems are related to the suboptimal solubility of the extracting solvent mixture. The use of unstable solvents is also a problem. Another challenge is the instability of curcumin to oxidation. In order to overcome these problems, Degot et al. [69] considered a promising alternative solvent medium, which is a ternary system consisting of water–sodium salicylate (anionic preservative hydrotrope)–ethyl acetate. Good extraction yields (~90% Soxhlet reference) can be achieved in the area of best solubility. Surprisingly, hydrophobic curcumin showed significant solubility in systems containing a significant amount of water in the presence of sodium salicylate. These mixtures are far from the breaking point but show a significant structure. The solubility is even higher than in pure ethyl acetate.

Curcumin was extracted from *Curcuma longa* L. using a green, biology-based, food-approved surfactant-free microemulsion (SFME) consisting of water, ethanol, and triacetin [70]. Due to the high solubility in the tested three-component mixtures, an attempt was made to prepare highly concentrated tinctures, in total up to ~130 mg/mL of curcuminoids in a solvent, through the repeated extraction of fresh rhizomes in the same extraction mixture. It was shown that the amount of water had a significant influence on the number of cycles that could be performed. In addition, the purity of the individual extracts was increased to 94% by performing several purification steps, e.g., vacuum distillation and freeze-drying. Additional stability tests in these studies showed that curcumin solutions can be stable for up to five months when hidden from natural light [70].

## 4. Beneficial Effects of Turmeric and Curcumin in the Treatment of Human Diseases

### 4.1. Alzheimer’s Disease

Demographic aging is a global process that comes with many challenges. Today, human life expectancy is much longer, resulting in a higher proportion of elderly people. Nevertheless, the incidence of Alzheimer’s disease (AD) and other dementias is increasing. Screening studies in mouse neuroblastoma cell models have been carried out to determine the link between the effects of curcumin and the course of Alzheimer’s disease and have shown that curcumin may play a role in alleviating AD pathology. The results provide new suggestive insights on the potential application of, inter alia, curcumin as a prophylactic and therapeutic agent against AD [71]. A study conducted in a rat model confirmed the effect of curcuminoids on gene expression, showing that each component of the curcuminoid mixture clearly influences gene expression, highlighting the therapeutic potential of curcuminoids in AD [72]. In another study in a rat model [73], α and β anomers of curcumin glycosides (CGs) were synthesized and evaluated for the treatment of Alzheimer’s disease. Both anomers led to a significant increase in the level of glutathione and acetylcholine and at the same time caused a decrease in the peroxidation of lipids and protein carbonyls in the brain tissue. This study revealed that CG compounds have enhanced antioxidant and anti-AD activity. These results support the fact that CGs, compared to donepezil as a commercial drug, can reduce the progression of Alzheimer’s disease with fewer side effects [73].

### 4.2. Rheumatoid Arthritis

Curcumin is known to have powerful anti-inflammatory and antiarthritic properties. A randomized, double-blind, controlled trial in a human model [74] evaluating the safety and efficacy of curcumin alone and in combination with diclofenac sodium in patients with active rheumatoid arthritis (RA) has shown that curcumin treatment is safe and is not associated with any adverse effects. This study provides evidence for the safety and superiority of curcumin treatment in patients with RA and highlights the need for future large-scale studies to validate these results in patients with RA and other joint diseases.

The effectiveness of curcumin is was emphasized in a previous study in which RA patients were given curcumin nanomicels three times a day for 12 weeks [75]. After that, it was found that the addition of curcumin nanomicels to RA patients’ medications led to positive changes.

### 4.3. Cancers

Currently, curcumin is gaining popularity as an anticancer agent [76]. So far, turmeric has been confirmed to have the ability to neutralize carcinogenic substances, block the cell cycle, multiply many types of cancer cells, and induce their apoptosis. Due to its ability to inhibit tumor growth at the stage of initiation, as well as inhibit the proliferation of malignant cells at the stages of promotion and progression of the neoplastic process, curcumin belongs to both groups, blocking and suppressive chemopreventive agents. Curcumin inhibits cancer growth both in vitro and in vivo. Its action leads to the inhibition of cell proliferation in various tumor cell lines and the inhibition of oncogenesis [77]. Experimental evidence reveals many molecular aspects, including transcription factors, cell cycle proteins, enzymes, cell surface adhesion proteins, and cytokines. Although curcumin has pronounced antitumor activity, its relatively poor stability has been highlighted as one of the major problems in therapeutic applications [78].

The development of cisplatin resistance is a common cause of cancer recurrence in colorectal cancer (CRC). The long noncoding RNA KCNQ1OT1 has been shown to exert its oncogenic function in cancers. Curcumin is a natural phenolic compound that can effectively suppress cisplatin resistance in CRC. In vivo studies confirmed that the ectopic expression of KCNQ1OT1 reverses the inhibitory effect of curcumin on the growth of cisplatin-resistant CRC cells [79].

Breast cancer is the most common malignant neoplasm in women in the world. It has been shown that curcumin exhibits antitumor activity against breast cancer and is less toxic than medicines [80]. Moreover, using it together with standard medical treatment can reduce the side effects of the therapy. Curcumin sensitizes cancer cells to cytostatic and enhances their action. In another example, curcumin was found to have therapeutic effects on colon cancer. Curcumin exerted anticancer effects by binding to core targets and interfering with colon cancer cell proliferation and apoptosis by regulating signal transduction pathways [81].

### 4.4. Liver Diseases

Studies have been conducted to evaluate the use of turmeric extract on hepatoma cells. Curcuminoids showed antitumor potential and induced the inhibition of cell growth through apoptotic changes. Therefore, it was concluded that substances from the *C. longa* L. rhizome (especially curcumin) can be used as a natural source of anticancer substances and can serve as a source of high-quality raw material for the pharmaceutical industry. This contributes to the scientific evidence for the use of this medicinal plant in traditional medicine [82].

Another disease associated with liver dysfunction is nonalcoholic fatty liver disease (NAFLD) [83,84]. It is one of the most common metabolic syndromes characterized by the accumulation of hepatic triglycerides (TGs) due to an imbalance between fatty acid uptake, synthesis, export, and oxidation. In a previous in vitro study [85], the effect of curcumin on a reduction in lipid accumulation in liver cells was investigated. These results indicate that the use of curcumin leads to the inhibition of lipogenesis in the liver and the antioxidant capacity. Curcumin has been found to exert a regulatory effect on lipid accumulation by reducing lipogenesis in hepatocytes. Therefore, curcumin extract may be active in the prevention of NAFLD [85].

### 4.5. Gastrointestinal Diseases

Inflammatory bowel disease (IBD) may result from mutations in genes encoding innate immunity, which, together with environmental factors, may lead to the exacerbation of inflammation [86]. The gastrointestinal microbiome, immune system, and barrier function contribute effectively to IBD, although its exact etiology remains unknown [87].

Curcumin has been shown to exert a therapeutic effect on IBD by targeting a spectrum of cellular and molecular pathways. Moreover, IBD leads to recurrent anemia, poor iron absorption, and reduced quality of life. Therefore, it is recommended to use complex curcumin as an anti-inflammatory agent. Moreover, curcumin is able to modulate iron metabolism proteins [88].

In an in vivo study in a mouse model [89], the authors hypothesized that, due to the high concentration of curcumin in the gastrointestinal tract after oral administration, it may exert a regulatory effect on the intestinal microbiota. The same study found that curcumin significantly affected several representative families in gut microbial communities, including *Prevotellaceae*, *Bacteroidaceae*, and *Rikenellaceae*. Given the pathogenic links between the gut microbiota and many diseases, current findings could help interpret the therapeutic benefits of curcumin [89].

### 4.6. Antidiabetes Activity

Curcumin exhibits numerous biological and pharmacological activities, including antidiabetic activity. It seems that the therapeutic benefits of curcumin can be mainly attributed to the enol ketone as a functional group and the presence of active methylene hydrogen [90,91].

Another compound similar to curcumin is thymol (THY). It is a vegetable essential oil that occurs naturally in the plants of *Zingiberaceae* and *Lamiaceae* families. THY, a type of monoterpene, has been reported to have various biological activities, including anti-inflammatory, antihyperlipidemic, and antioxidant activity [92,93]. Moreover, it is widely used as a preservation agent in cosmetics [94].

The results obtained in another in vivo study in a rat model [93] indicated that thymol has significant antihypoglycemic and antihypolipidemic effects in diabetic rats. In addition, an evaluation of various biochemical parameters showed that the levels of creatinine, low-density lipoprotein cholesterol, very low-density lipoprotein cholesterol, and liver-related enzymes such as aspartate aminotransferase and alanine aminotransferase were reduced in diabetic rats compared with controls. The results of these studies have brought new knowledge on the positive effect of thymol on diabetic complications in an animal model, which may translate into a better understanding of its disease in humans [93].

### 4.7. Urinary Route Infections

Urinary tract infections are one of the most common diseases in humans. Women are much more likely to develop these infections than men [95]. The reason is the presence of certain pathogens such as Escherichia coli, Proteus mirabilis, Pseudomonas aeruginosa, and Serratia marcescens. These uropathogens have a controlled quorum sensing (QS) capacity to form biofilms, leading to infections. In one in vitro study [96], curcumin was found to inhibit biofilm formation in species such as Escherichia coli, Pseudomonas aeruginosa, Proteus mirabilis, and Serratia marcescens, possibly by interfering with their QS systems. Treatment with curcumin was also found to attenuate QS-dependent factors such as exopolysaccharide production, alginate production, and uropathogen mobility. In addition, curcumin increases the susceptibility of the marker strain and uropathogens to conventional antibiotics [96].

### 4.8. Skin Diseases

In some countries, widespread inadequate sanitation and the lack of basic hygiene measures lead to the development of common fungal skin diseases. Environmental factors (high temperature and humidity) also play a significant role. Typically, these infections are named after the affected parts of the body. Tenia corporis is a dermatophyte infection of the epidermis, most often caused by species of the genera Trichophyton and Microsporum [97]. The infection is generally confined to the stratum corneum.

In a study in a human model [98] based on the evaluation of some essential oils, *Curcuma longa* L. showed the strongest antifungal activity, completely inhibiting the growth of mycelium caused by Microsporum gypseum and Trichophyton mentagrophytes. The oil also showed a broad fungicidal spectrum, inhibiting the growth of mycelium of other fungi, i.e., Epidermophyton floccosum, M. nanum, T. rubrum, and T. violaceum. At the end of treatment, 75% of the patients had completely recovered, while 15% showed significant improvement in their disease. Based on these results, an ointment containing compounds obtained from *Curcuma longa* L. can be used commercially after ongoing successful clinical trials [98].

### 4.9. Antibacterial Effect

The versatile use of curcumin has led scientists to elucidate its role in *Helicobacter pylori* infection. Based on research, it is predicted that curcumin may be a potential therapeutic agent against *H. pylori*-mediated gastric pathogenesis. Curcumin fulfills the features of an ideal chemopreventive agent against gastric carcinogenesis processes mediated by *H. pylori* [99,100].

The antimicrobial effect of curcumin is due to the formation of transmembrane pores or ion channels on the cell membrane, which leads to the leakage of essential metabolites and disrupts the structure of the bacterial cell wall, disrupting the flow of many important components that are responsible for the synthesis and maintenance of cell wall rigidity [101].

### 4.10. Curcumin versus Coronavirus

Turmeric has antiviral and anti-inflammatory properties, which can be beneficial for patients with COVID-19 [102,103]. Some have expressed concerns that curcumin might increase the expression of ACE2 and worsen COVID-19 infection as well as increase pro-inflammatory cytokines and worsen the course of illness in patients with cytokine storm. On the contrary, curcumin binds to the viral S protein and the viral attachment sites of the ACE2 receptor protein to inhibit the entry of SARS-CoV2 [104,105]. In addition, curcumin has been shown to reduce inflammatory cytokines in COVID-19 patients. Therefore, the use of curcumin in a clinical trial should be considered as a new treatment option [106]. The potential effects of curcumin intake by patients suffering from COVID-19 include the inhibition of virus entry into the cell, the inhibition of viral and viral protease encapsulation, and the modulation of many cells’ signaling pathways [107]. Interestingly, a SARS-CoV-2 virus infection map shows that Southeast Asian countries, which are the largest producers and consumers of curcumin, have a very low number of deaths caused by COVID-19 [108].

In another in vivo study, Saber-Moghaddam et al. [109] assessed the effectiveness of oral nanocurcumin in hospitalized patients with mild to moderate COVID-19. Most symptoms, including fever and shivers, rapid breathing, muscle aches, and coughing, resolved much faster. Moreover, SaO_2_ was significantly higher. No patient in the treatment group experienced a worsening of their infection during the follow-up period, but 40% of the control group did. Oral curcumin nanoformulation can significantly reduce recovery time in hospitalized COVID-19 patients [109].

## 5. Beneficial Effects of Turmeric and Curcumin in Dogs

### 5.1. Addition of Curcumin to Diet

Snacks that contain curcumin have been found to be beneficial for dogs’ health. In one study [110], in snacks that were produced for dogs from commercially canned meat, curcumin was added, homogenized, and offered to dogs twice a day. On the 15th day, the erythrocyte and hematocrit counts were higher in curcumin-fed dogs than in control dogs. Dogs fed curcumin had lower levels of leukocytes, neutrophils, lymphocytes, nitric oxide, plasma reactive oxygen species, lipoperoxidation, and protein carbonylation on the day than control dogs. The authors of that study found that curcumin in dog snacks stimulated the antioxidant system and consequently reduced oxidative responses, which is beneficial for animal health. Moreover, 30 mg of curcumin/dog/day reduced the leukocyte count, suggesting a mild anti-inflammatory effect [110]. Moreover, according to research, turmeric significantly alleviates the symptoms of food allergy and inhibits the levels of IgE and IgG1, which significantly reduce the symptoms of food allergy [111].

### 5.2. Osteoarthritis

Canine osteoarthritis is the leading cause of euthanasia. Osteoarthritis is manifested by an increasing degree of limitation of joint movement. The main symptoms are lameness, pain, and a reduced quality of life. However, access to safe and effective treatment for osteoarthritis remains problematic. In animal nutrition, palmitoyl glucosamine and curcumin are used. Currently, a co-micronized formula is available on the European market in which both substances are processed together to reduce their particle size and increase their absorption rate. Another study [112] looked at whether this preparation can relieve pain in the joints and improve their mobility. The results showed that the dietary supplement alleviated the experimentally induced paw swelling and inflammatory cell infiltration and reduced sensitivity to painful stimuli. In an osteoarthritis model, the supplement was found to protect articular cartilage from degradation and effectively counteract neuropathic pain. Locomotor function was restored by 45% after the administration of the selected supplement. The findings suggest that a dietary supplement containing palmitoyl glucosamine co-micronized with curcumin could help treat osteoarthritis [112].

Comblain et al. [113] conducted a randomized, double-blind, prospective, placebo-controlled study to evaluate the efficacy of a diet containing a mixture of curcuminoid extract, hydrolyzed collagen, and green tea extract (COT) on dogs suffering from osteoarthritis. Therefore, 42 dogs were randomly assigned to receive either the experimental diet or the same diet supplemented with COT for 3 months. After this time, there was a significant reduction in pain during manipulation in the COT group but not in the control group. Pain evolved during manipulation depending on diet. Three other parameters assessed by subjective veterinary judgment (lameness, pain on palpation, and joint mobility) showed no statistical difference. In the subjective opinion of the caregivers, pain intensity worsened in the control group but remained stable in the COT group. The ability to get up from a supine position was significantly improved in COT compared to the control group [113].

Similar properties have been found with the supplementation of combined green-lipped mussel extract, curcumin, and blackcurrant leaves in dogs. It has been shown that, in dogs suffering from OA, the administration of this combination significantly improved the severity of the disease [114]. As revealed in another study [115], the integration of palmitoyl glucosamine co-micronized with curcumin into the diet can maintain meloxicam-induced pain relief in dogs with severe OA chronic pain.

### 5.3. Degenerative Myelopathy

Canine degenerative myelopathy (DM), recognized as a spontaneous model of amyotrophic lateral sclerosis, is known as a late-onset progressive degenerative disease of the spinal cord. Due to the progressive nature of DM, many dogs are euthanized, resulting in limited information on the final stage of the clinical picture. One study [116] analyzed the long-term clinical course from diagnosis to natural death to deepen the understanding of the overall clinical picture of this disease. As curcumin has been administered in some cases, its therapeutic effects have also been studied. Forty Pembroke Welsh Corgi dogs with a definitive diagnosis of DM by autopsy and histopathology were included in this study. Information on the long-term clinical signs of DM was investigated using a questionnaire that was collected from dog caregivers. Urinary incontinence and respiratory distress were observed in most dogs, as was death associated with respiratory distress. In contrast, signs of brain stem dysfunction were found at the end stage in a minority of the dogs. While more research is needed with more cases, the results of this study suggest that curcumin administration is effective in slowing disease progression [116].

### 5.4. Antibacterial and Antivirus Properties

Currently, wound healing is becoming more difficult due to the increasing resistance of bacteria to antibiotics. One study [117] aimed to investigate the antibacterial potential of raw turmeric, nanoturmeric, and nonsteroidal anti-inflammatory medicines against multi-drug resistant (MDR) *Staphylococcus aureus* and *E. coli* isolated from animal wounds. Nanocurcumin showed higher antimicrobial activity against *S. aureus* and *E. coli* MDR than raw curcumin. NSAIDs in combination with an antibiotic also showed a synergistic effect in inhibiting bacterial growth. Among the assumed risk factors, ectoparasites, body condition, and the use of antibiotics showed a significant association. This study showed a higher incidence of *S. aureus* and *E. coli* MDR from wounds with a significant association of assumed risk factors as well as promising antibacterial effects of nanocurcumin, raw curcumin, and NSAIDs in combination with antibiotics [117].

Canine parvovirus type 2 (CPV-2) has high morbidity and mortality rates in canines. As revealed in a previous study, curcumin inhibited CPV-2 NS1 endonuclease via numerous hydrophobic interactions. These results suggest that adding among others curcuminoids to the diet can prevent CPV-2 infection [118].

### 5.5. Liver Regeneration

As reported by Kashaeva et al. [119], plant hepatoprotectors may have an influence on the liver tissue by preventing the destruction of cell membranes and stimulating regenerative processes in hepatocytes. All animals underwent comprehensive conservative therapy, including a plant hepatoprotector code-named GTPS-4, which contained purified milk thistle extract, dry extract of tansy, St. John’s wort, turmeric, birch leaves, and immortelle flowers. In this study, therapy was combined with the heat treatment of liver tissue to enhance repair regeneration. The best effect was obtained after combining herbal preparations and contact coagulation techniques. It has been shown that the performed comprehensive therapy significantly improves the condition of liver tissues and their regenerative abilities and normalizes the homeostasis indices. The inclusion of plant hepatoprotectors in complex conservative therapy is, therefore, a promising direction in the development of biotechnology in hepatology [119].

### 5.6. Anti-Inflammatory and Antipruritic Effects

The well-known medicinal plants *Curcuma longa* L. (turmeric) and *Slybium marianum* L. (silymarin) are gaining more and more interest due to their therapeutic anti-inflammatory and antipruritic effects. As reported in one study [120], the group of investigators hypothesized that a commercially available oral/topical solution containing a combination of *C. longa* L. and *Slybium marianum* L. significantly reduced the results of itching after a short-term (1 week of twice-daily use) oral treatment of dogs with atopic dermatitis (AD). Twenty-six dogs with AD were used in the study, and they had not been previously treated. This study confirms the potential benefit of silifort toothpaste in the short-term relief of itching. This method of treatment can replace immunosuppressive applications with an anti-inflammatory effect [120].

Curcumin has anti-inflammatory and antioxidant effects and is beneficial for humans with diabetes; however, data on its effects on canine diabetes are limited. Suemanotham et al. [121] showed that supplementation with curcuminoids as part of standard therapy significantly reduced oxidative stress along with an increased ratio of glutathione to oxidized glutathione. Curcuminoids improve insulin sensitivity and reduce complications associated with cardiovascular disorders.

In beagle dogs with natural periodontitis, curcumin was given orally, which significantly reduced the clinical signs of periodontitis and pro-inflammatory cytokines. These studies support the clinical potential of curcumin as a new additive in the treatment of chronic periodontitis [122].

### 5.7. Wound Healing

Some people, especially in the elderly age group, still prefer to use traditional and local methods rather than seeking medical facilities, and one of these methods is the application of turmeric powder and shrubs to cure dog bites [123]. An experimental exploration of traditional Asian medicine revealed the pharmacological effects of topical application of ghee. In that study [124], ghee taken from sheep fat was mixed with powdered rhizomes of *Curcuma longa* L., and its potential therapeutic effect was assessed on accelerating surgical wound healing; in addition, the study was carried out to compare the effects of *Curcuma longa*–ghee and hyaluronic acid on gingival wound healing in dogs after surgery. A significant difference was observed in the inflammatory and repair parameters of the healing process between the cases treated with this new preparation and the cases treated with hyaluronic acid. The obtained results indicate a positive potential therapeutic effect on the healing of surgical wounds, in particular, an improvement in the consequences of periodontal treatment after surgical procedures [124]. In another study, turmeric extract-loaded carboxymethyl cellulose/silk sericin dressings exhibited inhibitory effects on the growth of bacterial strains, which was found to be associated with the controlled release of the turmeric extract [125].

### 5.8. Others

The use of curcumin may also be effective in controlling ectoparasites in companion animals. Studies analyzing the ability of essential oils to protect against a tick bite by a common tick showed that dogs with turmeric oil sprayed on their hair were significantly less likely to attach the tick to their paws and abdomen [126].

Caregivers of dogs suffering from cancer especially pay attention to their proper nutrition [127]. Surveys show that caregivers of healthy dogs are more likely to feed them with commercial dry food, while home-cooked and raw diets are more common among dogs with cancer. The use of supplements, especially cannabidiol products, mushroom extracts, or turmeric/curcumin, is also more common in this group. Alternative diets and supplements are more popular with caregivers of dogs with cancer than among caregivers of healthy dogs [127]. An example of an aggressive cancer is canine osteosarcoma, comprising 85% of canine bone neoplasms. Curcumin analog RL71 has potent cytotoxic activity in canine osteosarcoma cells, triggering apoptosis at concentrations achievable in vivo [128].

Protective effects of curcumin on canine semen have been demonstrated against damage caused by cryopreservation procedures, including an improvement in sperm parameters, the protection of sperm against ROS, and an increase in the expression of the NADPH 5 (NOX 5) oxidase gene [129,130].

In general, recent studies have shown that curcumin not only stimulates the antioxidant system and reduces oxidative responses in dogs but also reduces leukocyte counts, suggesting a mild anti-inflammatory effect achieved in dogs fed 30 mg/dog/day [131].

## 6. Bioavailability, Disadvantages, and Hazards of the Use of Curcumin

Issues that significantly limit the effectiveness and usefulness of curcumin include its low bioavailability, attributed to its insolubility in water, and its rapid metabolism to metabolites that become inactive. Curcumin is a compound with good oil solubility but is practically insoluble at room temperature in water with an acidic and neutral pH. Although it is soluble in an alkaline environment, it is prone to self-degradation.

In order to increase the bioavailability of curcumin, the formulations used can be broadly classified according to a number of approaches. Curcumin is fat-soluble, so it is recommended to consume curcumin with a fatty meal to improve its absorption.

Curcumin is a highly reactive compound due to its unique molecular structure. It is sensitive to visible light and UV, breaking down into a number of compounds, the most important end products of which are vanillin and ferulic acid [132]. Uncontrolled and unknown exposure to light has cast a shadow of unreliability on curcumin research to date. Therefore, when researching curcumin, extra care should be taken to avoid exposure to light. Curcumin can also be oxidized by free radicals and oxygen radicals, and many of the degradation products are responsible for some of the well-known antioxidant properties of curcumin [133].

When curcumin is ingested, reactions take place that further reduce the delivery of curcumin to the target sites. In the gastrointestinal tract, curcumin tightly binds to the mucus, further delaying the uptake of epithelial cells and subjecting curcumin to auto-oxidation and oxidative degradation. After transport to epithelial cells, extensive biotransformation occurs [134].

Curcumin can be used as an ingredient in functional foods. However, the low bioavailability of curcumin after oral administration due to its poor water solubility, auto-oxidation, instability at neutral/alkaline pH, and active metabolism [135,136] hinders its use as a bioactive material for functional food products or pharmaceuticals. The low oral bioavailability of curcumin can be overcome by the use of absorption enhancers and formulation strategies.

In preclinical and clinical studies, piperine inhibited the activity of metabolic enzymes. However, the improvement in bioavailability obtained with enhancers, including piperine, did not reach a satisfactory therapeutic level. Formulation strategies, including, but not limited to, nanoparticles, nanoparticles with added curcumin/piperine, liposomes, and nanoemulsions, have been proposed to overcome the low bioavailability of curcumin [137,138,139,140,141].

In order to apply a bioactive compound in a high-quality medical context, an appropriate extraction technique is required. In order to select the appropriate one, various factors such as high yield, integrity, and selectivity for the target compound must be taken into account [142,143].

Despite the efficacy and safety of curcumin, its therapeutic potential is still questioned due to its relatively poor bioavailability in humans, even when administered at high doses [144]. In general, the oral bioavailability of curcumin is low due to its relatively low absorption in the small intestine combined with its significant reduction and conjugated metabolism in the liver and elimination via the gallbladder. Its poor bioavailability is also affected by the binding of curcumin to enterocyte proteins, which may modify its structure [145]. The bioavailability of nutraceuticals is a function strictly dependent on changes in the gastrointestinal tract [146].

The bioavailability of polyphenols in food sources may also depend on the origin, food processing, and macronutrients. Their levels and profile patterns depend not only on the type of raw material but also on environmental conditions such as climate, soil, and plant stress factors, as well as maturation and storage methods. Food processing, such as grinding, drying, and heating, changes the food matrix and the composition of the polyphenols. In addition, macronutrients, especially lipids, can affect the solubility and absorption of curcumin. Therefore, turmeric should be associated in cooking with ingredients rich in lecithin, such as eggs or vegetable oils, in order to increase the availability of curcuminoids [147]. In addition, adding powdered curcuminoids to buttermilk, prior to its preparation, results in the increased bioavailability of curcuminoids. The digestive steps also contribute to the low bioavailability of polyphenols, affecting the solubility, degradation in the gut environment, and the rate of penetration in the small intestine [148].

There is evidence to suggest that gender may have a significant influence on the pharmacokinetics of curcumin. These differences may be due to gender-specific factors such as higher body fat in women [149,150].

A natural product that can modify the distribution and bioavailability of curcumin is the previously mentioned piperine, a natural alkaloid of black pepper, which is a potent inhibitor of biotransformation, especially glucuronidation. The combination of curcumin and piperine resulted in a three-fold increase in bioavailability compared to pure curcumin [144]. Colloidal nanoparticles showed a 15-fold increase in the concentration of administered curcumin as a result of increased absorption from the gastrointestinal tract as a result of colloidal dispersion [151]. As reported by Aslam et al. [152], the association of meloxicam with curcumin in a biodegradable nanocarrier system could provide a promising antipyretic, antinociceptive, and anti-inflammatory therapeutic approach for acute conditions.

As mentioned earlier, curcumin is used for better wound healing. However, its bioavailability and therapeutic efficacy are quite low. One study [153] described the use of phospholipid complexes for wound healing. Curcumin–phospholipid complex (CPC) was prepared via solvent evaporation. In addition, CPC was loaded into an in situ hydrogel-forming poloxamer (ISG). CPC ISG showed a stronger wound-healing effect, especially in the early phase. Epidermal regeneration was significantly improved by CPC ISG compared to control [153].

To increase the bioavailability of curcumin, a proprietary liposome-encapsulated formulation was developed that allows for intravenous administration and has been shown to have the highest concentration in lung tissue. Another study [154] characterized the anticancer and antiangiogenic effects of Lipocurc in vitro, in addition to evaluating Lipocurc infusions in dogs with naturally occurring cancer. The effect of Lipocurc, compared with free curcumin, on the viability of canine osteosarcoma, melanoma, and breast cancer cell lines, as well as the ability of Lipocurc to inhibit endothelial cell viability, migration, and tube formation, were assessed. Tumor cell proliferation was inhibited by curcumin at concentrations exceeding those achievable in canine lung tissue. Likewise, the equivalent high concentrations of Lipocurc and curcumin also inhibited endothelial cell viability, migration, and tube formation. Four of the six dogs completing the scheduled Lipocurc infusions experienced stable disease; however, no radiological responses were detected [154].

To overcome the bioavailability problem, a study [155] developed nanoformulation using chitosan/alginate nanoparticles (CANPs) for diethyl curcumin disuccinate (CDD), which has antitumor activity against MDA-MB-231 human breast cancer cells and Caco-2 cells. The main disadvantage of CDD is its poor water solubility and low bioavailability in the gastrointestinal tract. In this study, CDD-loaded CANPs (CDD-CANPs) exhibited good stability after exposure to simulated digestive fluids and ultraviolet light, and a CDD sustained release profile was observed in simulated digestive and body fluids. Compared to free CDD, CDD-CANPs showed improved stability, bioavailability, bioavailability, cellular uptake, and cytotoxicity to HepG2 cells, which shows promise for cancer treatment [155].

In order to increase the bioavailability, a previous study investigated the production of ternary nanoparticles using the high amylose complex, stearic acid, and soy protein isolate as coating materials, as well as the encapsulation of curcumin in them [156]. An in vitro release test showed that the ternary starch molecules can control the stable release of curcumin in simulated intestinal fluid. This study offers a novel approach to high biomass encapsulation and sustained release of polyphenols [156].

To overcome bioavailability issues, Huynh et al. [157] formulated the curcumin solid dispersion (CSD) (to enhance curcumin solubility) and incorporated this solid dispersion in the floating tablet formula (to bypass the alkaline pH in the intestine). The tablets were optimized by varying numerous parameters, including the granulating solvent, the binder excipient, the type of gas-generating excipients, the ratio of gas-generating excipients, the type/amount of matrix generator, and the tablet hardness. The best formula possesses a short floating potential of 35 ± 1 s, a long floating retention time of >8 h, good tablet integrity during the floating duration, enhanced curcumin solubility/dissolution profiles to >200 times, and perfect physicochemical stability for at least 2 years in the normal storage condition. Moreover, the floating tablet incorporating the CSD could be a potential pharmaceutical product for stomach cancer treatment [157].

Athira et al. [158] characterized the preparation and evaluation of water-soluble octenyl succinylated starch–curcumin nanoformulation with increased bioavailability and anticancer potential. It was found that nanocurcumin loaded with starch octenyl succinic anhydride (OSA) has antitumor potential for HeLa cells. A significant increase in cellular uptake can also be achieved with OSA starch containing nanocurcumin. Pharmacokinetic studies showed that the bioavailability of nanocurcumin was increased by approximately 71% [158].

In studies conducted on beagle dogs, it was shown that curcumin inclusion complex β-cyclodextrin (CUR-β-CD), curcumin solid dispersion (CUR-PEG-6000), and curcumin phospholipid complex (CUR-HSPC) can effectively improve the bioavailability of curcumin, even by over 200% [159].

As mentioned earlier, curcumin has a few disadvantages, such as water insolubility, slow cell absorption, limited bioavailability, pH-dependent instability, rapid metabolism, and unclear clinical efficacy. These disadvantages limit its utilization as an effective therapeutic agent, and in particular, its activities were recently questioned and considered to be deceptive. Curcumin has been the subject of numerous studies to increase its stability and bioavailability. The topical use of curcumin may enhance its strength, pharmacological activity, solubility, and therapeutic efficacy [144,160,161,162,163]. Despite the many benefits of introducing turmeric and its main substance curcumin for the prevention and treatment of many diseases, it is also worth considering the associated risks. Chen et al. [164] studied the cytotoxic effect of curcumin on the blastocyst stage of mouse embryos, the subsequent attachment of the embryo, and in vitro and in vivo growth, through implantation via embryo transfer. Blastocysts treated with 24 µM curcumin showed significantly increased apoptosis and decreased total cell count. This was associated with a reduced implantation rate and increased resorption of post-implantation embryos in the mouse uterus. These results collectively indicate that in vitro exposure to curcumin triggers apoptosis and delays early post-implantation development when transferred to mice. In addition, curcumin induces apoptotic damage to mouse blastocysts by producing ROS and further promotes mitochondria-dependent apoptotic signaling processes, impairing further embryonic development [164].

Furthermore, turmeric is a relatively expensive raw material, and recently, its prices have been constantly fluctuating in the world [165]. So far, turmeric has been used, among others, as a raw material for the production of steroids, thanks to the diosgenin extracted from it. Currently, due to high prices as well as environmental pollution, the production of steroids is gradually moving toward various methods of microbial transformation [166].

In the food industry, a common problem is the adulteration of products, i.e., noncompliance with the manufacturer’s declaration in terms of the presence of given ingredients/substances. Ground spices can be adulterated with artificial colors, starch, chalk powder, etc., to enhance their appearance. High-value ground spices are often adulterated for economic purposes. Adulteration is difficult to identify based solely on visual and sensory data [167]. Turmeric is also vulnerable to this type of practice. To this end, various methods are being developed to detect intentional adulteration [168,169].

An example of another threat is the presence of insects in ground turmeric. The species found in this spice is, for example, the cigarette beetle (*Lasioderma serricorne*), which is responsible for damaging the spice. Insects not only feed on the spice but also contaminate it through excretions, molts, and remains of dead insects, which is undesirable at a commercial level. In addition to the damage caused by insects, their presence promotes the spread of fungal and bacterial diseases. Minj et al. [170] showed that the use of microwaves can help achieve maximum egg mortality while preserving the color of turmeric powder at different microwave power levels and different time intervals. Maximum lethality was observed at 550 W for 10 min and 650 W for 8–10 min.

Turmeric, like other raw materials, can be contaminated with heavy metals that accumulate in the body, leading to long-term health effects. In a study by Rahman et al. [171], samples were analyzed using atomic absorption spectroscopy (AAS). The concentration of most heavy metals was within the limits recommended by the World Health Organization (WHO) (6.0000 ppm), with the exception of lead (12.3469 ppm).

Another example is mycotoxins, which, like heavy metals, are a significant chemical threat, leading to long-term health consequences. It was shown that 24.2% of the spice samples were contaminated with aflatoxins, 11.7% of which were attributed to aflatoxin B1 (AFB1). The presence of aflatoxins in spices has been linked to sorting criteria, the handling of rejected spices, storage practices, awareness of spoilage causes, and awareness of aflatoxins in food [172].

## 7. Side Effects of Using Curcumin

The use of curcumin is gaining popularity in research and as a dietary supplement due to its low toxicity and the theoretical possibility of using it in high doses without significant side effects. However, taking curcumin in high doses can potentially cause side effects related to its ability to complex metals, mainly iron [173]. Currently, it cannot be ruled out that the long-term consumption of curcumin in various forms can cause anemia. On the other hand, higher iron levels are often associated with carcinogenesis, and the possibility of using iron chelators is being intensively studied in the therapeutic approach to cancer treatment, including in patients with gastric adenocarcinoma [174,175]. Curcumin can interact with many proteins, which may lead to both pharmacological benefits and may cause unwanted side effects such as the induction of gallbladder contraction (20–40 mg), decreased chemical sensitivity [176], and abdominal pain (200 mg) [177]. The most common side effects of curcumin are nausea and diarrhea [178]. Increased serum alkaline phosphatase and lactate dehydrogenase activity have also been reported [179,180]. Commonly reported side effects associated with the consumption of curcumin are gastrointestinal problems, i.e., abdominal pain, nausea, diarrhea, and gastrointestinal bleeding. For most people, curcumin’s gastrointestinal side effects are generally mild. However, these side effects may be more severe in patients suffering from ulcers [181,182].

On the other hand, as stated by Guest [183], curcumin is safe and has a good tolerability and toxicity profile in humans. Indeed, even high doses (up to 12 g/day) are well tolerated, and only minor side effects have been reported, for example, diarrhea. Hanai et al. [184] reported that the most common side effects of curcumin were related to gastrointestinal problems, such as nausea and the sensation of abdominal distension; they were usually mild and transient, and no subjects in the studies dropped out because curcumin’s side effects [185,186]. Other side effects include temporary tongue staining and a mild yellowish discoloration of the teeth that disappeared with brushing [187,188].

In animal studies, after the administration of high doses of curcumin, side effects such as increased liver weight, facial discoloration, and epithelial hyperplasia of the cecum and colon were observed [188]. Balaji and Chempakam [189] analyzed turmeric compounds for toxicity using in silico models. Screening 200 turmeric compounds based on their toxicity, including bacterial mutagenicity, carcinogenicity in rodents, and hepatotoxicity in humans, yielded 16 compounds with these characteristics. Out of the 200 compounds, 184 compounds were found to be toxic, 136 compounds were mutagenic, 153 compounds were carcinogenic, and 64 compounds were hepatotoxic.

## 8. Conclusions

Turmeric is a wild plant found in tropical countries. It is used as a spice and as a food coloring. The biologically active factors that give turmeric its extraordinary properties and color are curcuminoids. They are a group of substances that include curcumin, demethoxycurcumin, and bis-demethoxycurcumin. Curcumin is the dominant component, and it is also considered the most valuable of all curcuminoids. These substances exhibit pleiotropic effects, i.e., they have the ability to act in many directions on the bodies of both humans and dogs. In conclusion, this review shows that the most important pro-health effects observed after the administration of curcuminoids for humans and dogs include anti-inflammatory, anticancer, and antioxidant effects. Curcuminoids have also been shown to be effective in the treatment of metabolic disorders as well as central nervous system disorders. Positive results were particularly observed in models of neurodegenerative diseases such as Alzheimer’s or Parkinson’s disease; however, positive effects were also noted in patients with depression. The limitation in the use of curcumin is primarily associated with the low bioavailability of curcuminoids and poor water solubility. Common sense is necessary because, in many cases, its excessive use is harmful. Curcumin used in high doses may cause side effects, leading to gastrointestinal problems and even anemia. When consuming turmeric, one should bear in mind that, like other raw materials, there is a risk of contamination with chemical and biological hazards that may negatively affect the health of consumers.

Future research should focus on the use of curcumin as an adjuvant therapy for many diseases in companion animals. An important problem also seems to be the bitter taste of turmeric, which should be extensively analyzed in the case of, e.g., dogs.

## Figures and Tables

**Table 1:** Major nutrients of dried rhizomes of *Curcuma longa* L.

Item	Value (g/100 g)				
	[9]	[10]			[11]
Moisture (%)	8.92	9.05	8.90	9.01	8.00
Crude protein	10.32	9.23	8.32	7.34	11.73
Lipids	7.52	8.58	9.22	7.16	5.54
Crude ash	3.13	7.20	7.21	7.55	9.24
Crude fiber	5.05	8.52	7.82	8.41	3.26
Total carbohydrates	73.98	66.47	67.43	69.53	70.23

**Table 2:** Essential amino acid composition of turmeric.

Item	Concentration (g/100 g of Protein)		
	[8]	[14]	[15]
Arg	6.55	4.59	2.48
His	4.76	1.80	1.80
Ile	2.85	3.69	7.58
Leu	2.65	7.09	2.53
Lys	3.85	3.29	12.73
Met	1.50	0.40	3.28
Phe	5.75	4.69	nd
Thr	3.57	4.39	2.87
Trp	1.79	nd	nd
Val	4.75	4.19	1.53
Σ EAA	38.02	27.74	34.80

nd—no data.

**Table 3:** Macroelement content in dried turmeric rhizome.

Item		Value (g/100 g)	
	[14]	[17]	[18]
Ca	0.2294	0.1959	0.0082
P	nd	0.5917	0.0001
Na	0.1826	0.0194	0.0001
K	2.8780	1.4223	0.0017
Mg	0.2826	0.4545	0.0002

nd—no data.

**Table 4:** Microelement content in dried turmeric rhizome.

Item	Value (mg/100 g)		
	[14]	[17]	[18]
Cu	nd	0.18	nd
Zn	nd	0.25	22.90
Fe	0.02	7.93	2.40

nd—no data.

**Table 5:** Vitamin content in dried turmeric rhizome [14].

Item	Value (g/100 g)
B1	0.148
B2	0.166
B3	nd
B6	0.010
A	nd
C	nd
D	0.008
E	nd

nd—no data.

**Table 6:** Curcuminoids in dried turmeric rhizome.

Item	Value (g/100 g)		
	[20]	[21]	[22]
Curcumin	7.35	7.91	7.20
Demethoxycurcumin	3.10	3.08	3.00
Bisdemethoxycurcumin	3.08	3.20	2.90

**Table 7:** Phytochemical composition of dried turmeric rhizome.

Item	Value (g/100 g)		
	[9]	[28]	[29]
Alkaloids	0.76	6.64	8.46
Saponins	0.45	2.30	0.95
Tannins	1.08	nd	19.10
Flavonoids	0.40	8.00	13.42
Total phenols	0.08	4.91	36.67

nd—no data.

**Table 8:** Essential oils in dried turmeric rhizome.

Specification	Concentration (g/100 g)		
	[37]	[38]	[39]
Ar-tumerone	61.79	21.00	nd
Curlone	12.48	nd	7.59
Ar-curcumene	6.11	1.90	11.42
Zingiberene	2.98	2.60	5.13
α-Sesquiphellandrene	2.81	2.40	10.44
α-Bisabolene	1.48	0.40	3.04

nd—no data.

## Data Availability

Not applicable.
